# Breast Cancer and Resilience: The Controversial Role of Perceived Emotional Intelligence

**DOI:** 10.3389/fpsyg.2020.595713

**Published:** 2020-12-15

**Authors:** Rocio Guil, Paula Ruiz-González, Ana Merchán-Clavellino, Lucía Morales-Sánchez, Antonio Zayas, Rocio Gómez-Molinero

**Affiliations:** ^1^Department of Psychology, Faculty of Education Sciences, University of Cádiz, Puerto Real, Spain; ^2^INDESS (Research Universitary Institute for Sustainable Social Development), University of Cádiz, Jerez de la Frontera, Spain

**Keywords:** breast cancer, resilience, perceived emotional intelligence, emotional attention, emotional clarity, mood repair, mediation analysis

## Abstract

Cancer is a chronic disease that causes the most deaths in the world, being a public health problem nowadays. Even though breast cancer affects the daily lives of patients, many women become resilient after the disease, decreasing the impact of the diagnosis. Based on a positive psychology approach, the concept of co-vitality arises understood as a set of socio-emotional competencies that enhance psychological adaptation. In this sense, emotional intelligence is one of the main protective factors associated with resilience. However, it is not always as beneficial as it seems, and can lead to collateral effects on psychological adjustment. Given this controversy, this study aims to find the specific processes through which the dimensions of Perceived Emotional Intelligence (PEI) (Emotional Attention, Emotional Clarity, and Mood Repair) can act as a risk or protective factor in the development of resilience. The total sample was 167 women (Age: *M* = 43.26; *SD* = 12.43), 46.7% were breast cancer survivors, and 53.3% were healthy controls. The selection of women with breast cancer carries out randomly, recruited through the Oncology Units. The sample completed measures of resilience and PEI, through Resilience Scale (Wagnild and Young, 1993) and TMMS-24 (Salovey et al., 1995). The results showed that breast cancer survivors showed higher age and greater levels of resilience and mood repair than healthy women. The mediation analysis revealed that breast cancer survival and PEI predicted 28% of the variance of resilience. The direct effects showed that emotional clarity and mood repair increased resilience levels. Although breast cancer did not predict resilience directly, it does through mood repair by an indirect process. Besides, the analysis showed that emotional attention played a role in vulnerability, decreasing mood repair, and resilience. These research support theories that point to a possible dark side of PEI, thus, a great level of emotional attention makes dark the positive effect of mood repair and personal growth if a clear perception of emotions does not complement it. These results provide empirical support concerning the need to work complementary each dimension of PEI to avoid unwanted effects on intrapersonal adjustment.

## Introduction

Cancer is one of the most significant diseases due to its high incidence and morbidity, being one of the leading causes of death worldwide (World Health Organization, 2014). According to the European Cancer Information System (ECIS) (2018) in Spain was diagnosed with 32.825 breast cancer cases in 2018, standing for 30% of cancer diagnosed in women.

Despite survival rate increases, it is well known that breast cancer diagnosis, as well as the treatments derived from the disease, affects many vital facets (workability, interpersonal relationships, body image, or daily habits) assuming an important influence on physical and psychological well-being (Guil et al., 2016; Marroquín et al., 2016; Molano-Tobar and Varela, 2017; Catherine et al., 2019).

Nevertheless, recent research in the positive psychology approach has focused on psychological adaptation, suggesting that breast cancer survivors can cope and adjust to extremely unfavorable situations (Deshields et al., 2016). Although cancer diagnosis involves personal suffering, many breast cancer women can develop an ability to resist and accept life crisis, resulting in greater resilience and personal growth (García-Maroto Fernández et al., 2015; Stanton and Bower, 2015; Padilla-Ruiz et al., 2019; Ruiz-González et al., 2019), what in turn, decreasing the negative impact associated with the disease (Zayas et al., 2016; Gallagher et al., 2019). Resilience is a characteristic of the personality that moderates the negative effect of stress or adverse situations, thus promoting the adaptation process, involving emotional strength, courage and adaptability, among the characteristics are perseverance, having purposes in life, believe in oneself (Wagnild and Young, 1993).

Although these women adapt adequately to the disease process through resilience, it is considered, and we will see it throughout the text, that a useful skill or adequate levels to manage emotions can improve, even more, the adaptive process to disease. In this line, the concept of “co-vitality” arises in opposition to “comorbidity,” thereby referring to the set of personal factors, mainly socio-emotional competences, which are capable of increasing people's psychosocial adjustment (Furlong et al., 2014; Pérez-González et al., 2020). Thus, Deshields et al. (2016) understand resilience as a dynamic process influenced by emotional intelligence, among other personal and social risk and protection factors.

According to Salovey and Mayer (1990), emotional intelligence is defined as “the ability to perceive emotions, to access and generate emotions to assist thought, to understand emotions and emotional knowledge, and to reflectively regulate emotions to promote emotional and intellectual growth” (p. 5). However, recent research also explores people's beliefs about their emotional intelligence, termed as PEI (Salovey et al., 1995). Likewise, PEI refers to the self-perception of our ability to attend (Emotional Attention), discriminate (Emotional Clarity), and to regulate feelings and emotions (Mood Repair), to maintain adequate levels of well-being (Petrides et al., 2016).

This approach would be encompassed within the trait models, which consider EI as stable personality traits, behavioral tendencies and self-perceived abilities (Petrides, 2010). Even so, other models have emerged that attempt to conceptualize EI from different perspectives (Mestre and Fernández-Berrocal, 2007). They can be classified, also as skill models and mixed models. Skill models view EI as the ability to process emotional information to improve and guide thoughts. However, mixed models are incorporated as a third perspective that conceptualizes EI as a set of traits and competencies (Miao et al., 2017).

Studies focused on emotional intelligence generally address this construct as a protective factor in health, suggesting that the way that breast cancer women regulate their emotions influences their quality of life and enhance disease adaptation (Hermosilla-Ávila and Sanhueza-Alvarado, 2015; Brandão et al., 2016; Amirifard et al., 2017; Baudry et al., 2018). Besides, some studies show how emotional intelligence predicts changes in psychological, social and physical well-being, satisfaction, optimism, social adjustment and the quality of interpersonal relationships, as well as in post-traumatic growth in breast cancer women (Rey et al., 2013; Mahdavi et al., 2015; Ahoiee et al., 2017; Rider Mundey et al., 2019).

However, in recent years it has been shown that the belief of having the ability to handle our emotional ones facilitates this adaptation process (Schmidt and Andrykowski, 2004; González et al., 2010; Resurrección et al., 2014), what in turn, involves a faster recovery (Markovitz et al., 2015; Burga and Sánchez, 2016; Brandão et al., 2017; García Monzón and Navarro Machado, 2017). In this sense, previous research confirms that the perceived ability of women with breast cancer to moderate the negative affect caused by their disease contributes to improving adaptation and psychological well-being (Guil et al., 2016; Rider Mundey et al., 2019; Zayas et al., 2019). However, it seems to vary with age because of life experience (Fernández-Berrocal et al., 2012).

Despite this evidence, there are situations in which the promotion of emotional intelligence could cause maladaptive results for an individual. It has been demonstrated that high levels of emotional intelligence have been associated with intrapersonal and interpersonal side effects such as psychological ill-health, stress reactivity, emotional manipulation, or antisocial behavior. In this sense, there is a current debate about the contexts in which PEI could be beneficial or harmful (Davis and Nichols, 2016).

Besides, attending to the dimensions of PEI, not all of them contribute positively to psychological adjustment. Specifically, the ability to discriminate emotional states and to repair them properly has been associated with better health outcomes (Guil et al., 2019). However, paying too much attention to emotional states increases reflective thoughts, negative affective states, and even limit the development of psychosocial competencies (Extremera and Fernández-Berrocal, 2002, 2006; Lizeretti et al., 2012; Guil et al., 2019). There is even empirical evidence that certain therapeutic practices with an attentional component such as meditation and mindfulness can generate side effects that should be studied in greater depth (Cebolla et al., 2017). Nevertheless, if the attention is accompanied by the ability to discriminate and repair negative mood, these negative consequences are reduced, leading to better physical and psychological function (Cejudo et al., 2016; Gómez-Baya and Mendoza, 2018; Merchán-Clavellino et al., 2019, 2020).

Given the previous controversy about PEI, it is necessary to understand the processes through which emotional attention, emotional clarity, and mood repair could act as a risk or protection factor in the development of resilience in breast cancer survivors. Since the influence of these factors on resilience could be of great importance to improve psychological adjustment after suffering from breast cancer.

Therefore, the purpose of this study is, on the one hand, to analyze the differences that exist in emotional intelligence and resilience between breast cancer survivors and those who have not suffered from it. It is expected that women who have suffered from this disease, compared to those who have not to present better emotional intelligence and greater resilience. On the other hand, this study aims to explore how PEI (Attention, Clarity, and Emotional Repair) can mediate the relationship between breast cancer and resilience. It is hypothesized that breast cancer survivorship is associated with greater resilience and that these levels of resilience will be even better if associated with better emotional clarity and/or repair. At the same time, another pathway could occur whereby high levels of emotional attention would be associated with lower levels of resilience.

## Materials and Methods

### Participants

Study participants were 167 Spanish women with an age range between 18 and 69 years (*M* = 43.26; *SD* = 12.43). The total sample indicated that 52.7% were married, 31.7% were single, 7.2% were common-law partners, 5.4% were divorced, and 3% were widows. Regarding the work status, 34.1% were employed workers, 23.4% were unemployed, 15.6% were pensioners, 14.4% were housewives, 7.8% were on sick leave, and 4.8% were self-employed.

The participants were divided into two groups. Group “Breast cancer survivors” was composed of 78 breast cancer survivors (46.7%) and Control Group were 89 women without any oncological disease (53.3%). The common inclusion criteria for both groups were: (1) To be a woman; (2) To have more than 18 years; (3) To have an adequate cognitive ability to understand the psychological tests presented; (4) To not receive at the time of assessment, psychiatric and/or psychological treatment; and (5) To not be under the influence of psychoactive medication. Besides, for the breast cancer survivors group was taken into consideration: (1) To have been diagnosed with breast cancer at least 1 year before to participate in the study or to have received the medical discharge.

### Procedure

Breast cancer survivors were randomly recruited from the Oncology Units of the reference hospitals of the province of Cadiz, Spain. They were recruited with the help of doctors from the oncology units, women who met the participation criteria were informed about the study, and all the people who showed their interest were put in contact with the research team. Thus, finally, they were contacted, and they met individually.

Something similar was done with Control Group. In this case, the recruitment was carried out through social networks and the general population. Women in Group 2 were selected with similar socioeconomic and educational characteristics than Group 1 patients.

To carry out this study approval was obtained from the Ethics Committee of the referral hospitals (project identification code: PIN0109-2018). Prior participation, they had to sign an informed consent. Data collection was performed through paper-and-pencil questionnaires and were analyzed according to guidelines of the Declaration of Helsinki of the World Medical Association (WMA) (64th General Assembly, Fortaleza, Brazil, October 2013).

### Instruments

#### Resilience Scale

Wagnild and Young Resilience Scale (Wagnild and Young, 1993). It is a Spanish version adapted by Novella (2002). This instrument assesses the ability to face adverse situations. The scale is composed of 25 items on a Likert scale, ranging from 1 (totally disagree) to 7 (totally agree). The scale interpretation was ranged as follows: Non-resilience (25–74 points); Low resilience (75–100 points); Average resilience (101–125 points); High resilience (126–150 points), and very high resilience (151–175 points). Besides the global resilience measure, it includes six dimensions. Equanimity refers to a balanced perspective of one's life, meaningfulness is the ability to understand life purpose, perseverance attends to the ability to keep going despite adversity, existential aloneness is recognition of one's unique path, and self-reliance refers to the ability to believe in oneself. For the purpose of this study, only global resilience was included. Cronbach's alpha was 0.92.

#### Trait Meta-Mood Scale –TMMS-24-

Salovey et al. (1995), the Spanish version by Fernández-Berrocal et al. (1998), is a self-report instrument that assesses self-perceptions of emotional competencies. TMMS-24 comprised 24 items that assess three dimensions of emotional intelligence through eight items each one: Emotional Attention refers to perceived attention paid to own mood and emotions; Emotional Clarity denotes the perceived capacity to understand and discriminate emotional states; and Mood Repair refers to the subjective ability to regulate negative emotions in order to maintain emotional balance (Salovey et al., 1995). Responses were given on a Likert scale from 1 (completely disagree) to 5 (totally agree). Greater scores of dimensions indicate higher EI levels. In the present study, Cronbach's alpha for each of the dimensions was 0.86, 0.93, and 0.90 for emotional attention, emotional clarity, and mood repair, respectively.

#### Data Analyses

Preliminary analyses were carried out to compute descriptive statistics and internal consistencies for measures through Cronbach's alpha. Hence, we compared mean scores of both groups (breast cancer survivors and control group) by performing an analysis of variance, including Cohen's *d* to check the effect size, considering as follows: small effect (≤0.20), medium effect (between 0.21 and 0.5) or large (>0.50). These analyses were achieved with the statistical software SPSS 22 (IBM Corp, Chicago, IL).

Finally, a serial multiple mediation analysis was performed to test whether emotional attention, emotional clarity and mood repair mediated the relationship between breast cancer survivorship and resilience after controlling for the influence of age. So in this study, breast cancer survivors cancer was considered the first variable, as a binary variable (0 = control group and 1 = breast cancer survivors group) (predictor, X) and resilience as the outcome (Y). Emotional Attention (M1), Emotional Clarity (M2), and Emotional Repair (M3) were considered the mediator variables, and (e) was age as a covariate. This total effect was the sum of all possible pathways from X to Y, direct (c′), or mediating pathways (the indirect effects). The direct effect (c′) refers to the relationship between survival to breast cancer and resilience, after controlling for the mediators. The indirect effects are: the total indirect effect (a) represents the association between the predictor and the three mediators (a1, a2, and a3); the total indirect effect (b) refers to the role of the three mediators about resilience (b1, b2, and b3); the total indirect effect (d) refers to the relationship of the three mediators to each other (d21, d32, and d31), and the specific indirect effect (a1b1, a2b2, and/or a3b3) refers to the role of a specific mediator in the relationship between breast cancer survival and resilience. Furthermore, finally, age covariate (e) is established with each mediator (M) and result variable (Y). All mediation analyses described below were estimated with the PROCESS macro (Hayes, 2013) using SPSS 20 software. We used Model 6 to examine the direct and indirect effect (Hayes, 2013). We tested this model by using the bootstrapping approach (Preacher and Hayes, 2008). As criteria for statistical significance were used a 95% corrected bias and direct and indirect effect confidence interval set to 10.000 reiterations. They are considered statistically significant if zero is not included within the interval.

## Results

### Preliminary Analysis

Table 1 shows reliability coefficients, descriptive statistics for the total sample, and breast cancer survivors and women without breast cancer separately, as well as the range of variable values. The average levels show that the total sample had high resilience levels and adequate scores in all dimensions of PEI.

**Table 1:** Descriptive statistics for the total sample, breast cancer women, and women without breast cancer, range, and Cronbach's α.

	**Total sample**				**Breast cancer survivors**		**No breast cancer**	
**Variables**	**Range**	**α**	***M***	***SD***	***M***	***SD***	***M***	***SD***
Age	18–69		43.26	12.43	50.42	8.35	36.99	12.02
Resilience	67–174	0.92	139.69	20.19	144.04	17.45	135.88	21.69
EA	8–40	0.86	24.85	6.58	24.13	6.72	25.48	6.42
EC	11–40	0.93	29.04	6.96	29.42	6.98	28.71	6.96
MR	11–40	0.90	29.44	6.81	31.10	6.86	28.00	6.46

*EA, Emotional Attention; EC, Emotional Clarity; MR, Mood Repair*.

Concerning comparison in variables by groups, the analysis of variance (ANOVA) indicated statistically significant differences in age [*F*_(1, 165)_ = 68.24, *p* < 0.001, *d* = −13.43], resilience [*F*_(1, 165)_ = 7.04, *p* < 0.01, *d* = −8.16], and mood repair [*F*_(1, 165)_ = 9.06, *p* < 0.01, *d* = −3.10] showing that breast cancer women exhibited greater levels of these variables, with a high effect size. Nevertheless, there were no statistically significant differences in emotional attention [*F*_(1, 165)_ = 1.77, *p* > 0.05], and emotional clarity [*F*_(1, 165)_ = 9.0, *p* > 0.05].

As shown in Table 2, Pearson's correlations indicated positive associations between breast cancer survivorship with age, resilience, and mood repair. Besides, resilience was found to be positively related to emotional clarity and mood repair. Age was significantly and positively related to resilience and negatively associated with emotional attention. Moreover, we found positive associations between emotional attention and emotional clarity and emotional clarity with mood repair.

**Table 2:** Pair-wise correlations between the all study variables.

**Variables**	**1**	**2**	**3**	**4**	**5**	**6**
1. Age	–					
2. Breast cancer	0.54^**^	–				
3. Resilience	0.20^**^	0.20^**^	–			
4. EA	−0.20^**^	−0.10	−0.15	–		
5. EC	0.04	0.05	0.35^**^	0.28^**^	–	
6. MR	0.09	0.23^**^	0.48^**^	−0.07	0.56^**^	–

^*^p < 0.05;

** *p < 0.01*.

The statistically significant correlations between all the study variables in each group are described below (*p* > 0.05). Correlations between emotional clarity (*r* = 0.331) and mood repair (*r* = 0.382) with resilience are observed for the control group and the dimensions of emotional intelligence are correlated in such a way: attention with clarity (*r* = 0.307) and clarity with repair (*r* = 0.648). The same relationships are shown in the cancer group; emotional clarity (*r* = 0.367) and mood repair (*r* = 0.566) with resilience and attention with clarity (*r* = 0.261) and clarity with repair (*r* = 0.470).

### Mediation Analysis

As shown in Table 3, it is confirmed that breast cancer survivorship explained only 5% (*R*^2^ = 0.05) of the variance of resilience being non-statistically significant. However, when the PEI dimensions were included in the model, the explanatory ability of the entire model was increased to 28% (*R*^2^ = 0.28) in a statistically significant way.

**Table 3:** Serial Mediator Model: Model summary, total effect, direct effect, and indirect effect.

**Model summary**	***R*^**2**^**	**MSE**	***F***	**df1**	**df2**	***p*** **(sig)**	
**Total effect model**	0.28	300.46	12.82	5.00	161.00	0.00	
Breast cancer on resilience	0.05	390.60	4.57	2.00	164.00	0.14	
					**95% CI**		
	**Path**	**Coefficient**	**SE**	***T***	***p***	**BootLL**	**BootUL**
**Direct effects**							
Age on EA	e_M1_	−0.11	0.05	− 2.30	0.02	−0.21	−0.02
Breast cancer on MR	a_3_	3.03	0.98	3.08	0.00	1.08	4.96
EA on resilience	b_1_	−0.47	0.23	−2.07	0.04	−0.92	−0.02
EC on resilience	b_2_	0.55	0.25	2.18	0.03	0.05	1.06
MR on resilience	b_3_	1.02	0.26	3.99	0.00	0.51	1.52
EA on EC	d_21_	0.31	0.08	3.88	0.00	0.15	0.47
EA on MR	d_31_	−0.25	0.07	−3.68	0.00	−0.38	−0.11
EC on MR	d_32_	0.60	0.06	9.69	0.00	0.48	0.72
**Indirect effect**							
Ind_3_ via MR	a_3_b_3_	3.09	1.26			1.08	6.23

Regarding the direct effects, we found positive predictive associations of breast cancer survivorship on mood repair (a_3_: β = 3.03, *p* = 0.00), emotional clarity and mood repair on resilience (b_2_: β = 0.55, *p* = 0.03; b_3_: β = 1.02, *p* = 0.00, respectively), emotional attention on emotional clarity (d_21_: β = 0.31, *p* = 0.00), and the latter with mood repair (d_32_: β = 0.60, *p* = 0.00). Besides, other statistically significant direct effects indicated a negative association between age and emotional attention (e_M1_: β = −0.11, *p* = 0.02), and emotional attention with a decrease in mood repair (d_31_: β = −0.25, *p* = 0.00), and resilience (b_1_: β = −0.47, *p* = 0.04).

Although emotional clarity and mood repair are favorably related to each other and resilience, it does not occur with emotional attention. It is here where is observed the true dark side of PEI.

**Figure 1:**
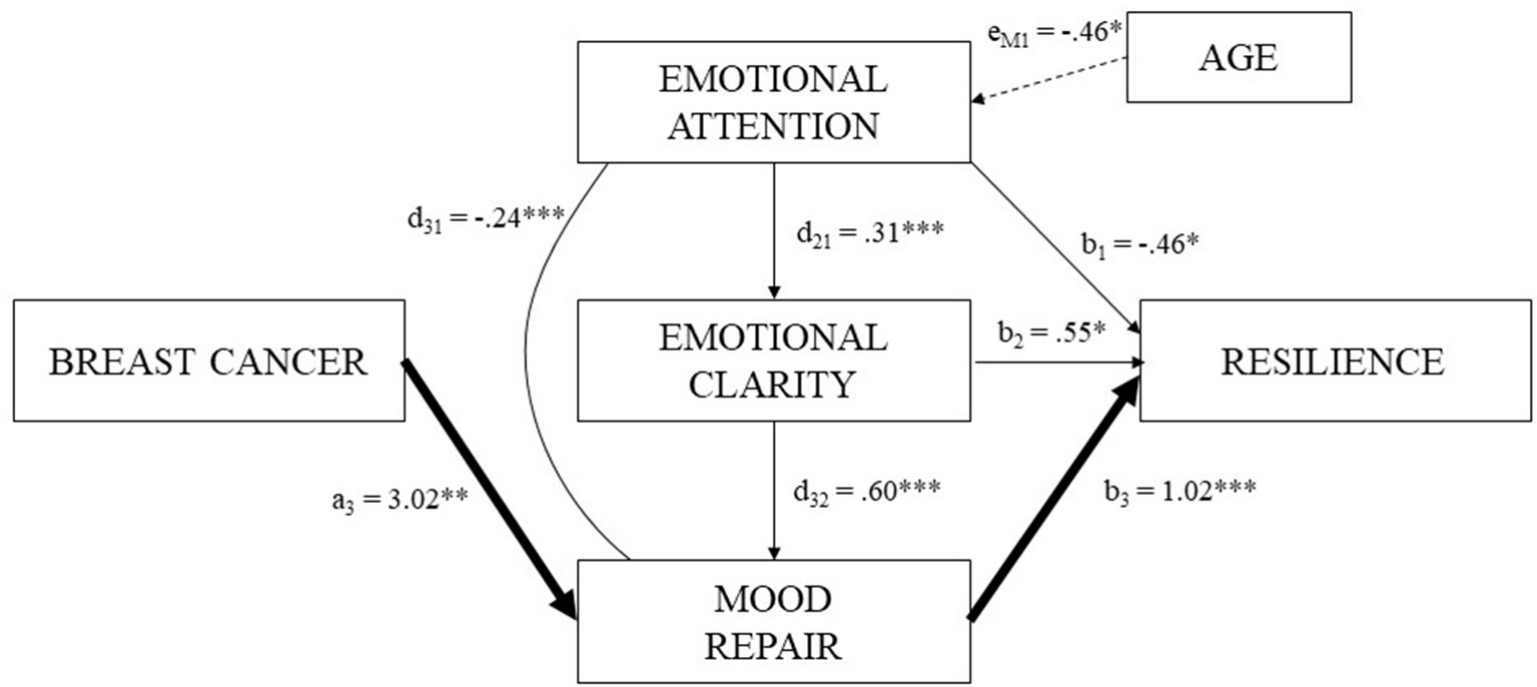
Serial multiple mediation model: direct and indirect effects among breast cancer, resilience and PEI.

In respect of indirect effects, only indirect effect 3 (a_3_b_3_; 95% bias-corrected CI) showed statistical significance in the model. Thus, overcoming breast cancer was associated with a greater ability to repair adverse emotional states, which in turn increased women's resilience (β = 3.09, SE = 1.26, 95% CI: 1.08, 6.23). In this case, a full mediation effect was found because the variation in resilience is explained only through the mediator, specifically mood repair, and not by the direct effect of breast cancer survivorship. The diagram of the model is summarized in Figure 1.

## Discussion

To date, there is evidence of a positive association between emotional intelligence in the oncology field. Nevertheless, there is no literature about the specific processes through which the PEI dimensions can act as a risk factor in the development of resilience. Hence, this paper provides empirical evidence about the measuring effect of PEI and how it should be enhanced in health programs.

Descriptive statistics indicated that the total sample showed adequate levels of resilience and all dimensions of PEI. Our initial hypothesis is confirmed because there are differences in means between both groups, according to the diagnosis of cancer, with statistically significant differences in the levels of age, resilience, and mood repair. In this sense, breast cancer survivors showed higher levels of these variables, without differentiation in levels of emotional attention and emotional clarity. In other words, while both groups have no statistically significant differences regarding their perceived ability to express and identifying feelings, women that have been under the traumatic experience of breast cancer, perceive themselves more competent regulating their moods and emotions. Our results are in line with previous studies in which women who suffer or have had breast cancer are associated with different types of strength indicators, such as resilience, concerning women who have not experienced breast cancer (García-Maroto Fernández et al., 2015; Stanton and Bower, 2015; Padilla-Ruiz et al., 2019; Ruiz-González et al., 2019).

Therefore, our findings encourage theories that highlight the changes that this illness entails, such as transformations in the way to face traumatic situations (Deshields et al., 2016; Gallagher et al., 2019). The existence of a statistically significant positive correlation between breast cancer survivorship with resilience and mood repair pointed out that women who overcome with the disease are more strength by the illness and repair better their moods (Amirifard et al., 2017; Brandão et al., 2017; García Monzón and Navarro Machado, 2017). Moreover, as mention in the introduction section, PEI is related to resilience. Hence, our study suggests that the ability to discriminate emotional states and regulate negative affective states is positively associated with personal growth after experiencing adverse situations (Burga and Sánchez, 2016).

Regarding the mediation model, the direct effects showed that greater resilience is predicted through the increases in the levels of both emotional clarity and mood repair. The hypothesis established in this study is confirmed. Hence, our results reinforce research that demonstrates that women who perceive their emotions clearly and trust in their abilities to repair their emotional states are more strengthened and resilient (Cejudo et al., 2016). Our results also showed that age reduces the attention paid to emotions, showing that all the changes associated over time modify how people handle the emotions derived from them (Fernández-Berrocal et al., 2012).

Moreover, the ability of breast cancer survivors to accept adversities giving a positive meaning is predicted by the effect of repairing their emotional states through an indirect process. These finds are in line with studies that affirm that, although breast cancer involves adverse emotional reactions, also increases the capacity to repair them effectively, leading to personal growth (Markovitz et al., 2015; Deshields et al., 2016; Marroquín et al., 2016).

Even though emotional intelligence appears to be a health-promoting factor (Pérez-González et al., 2020), our study provides new insights into the dark side of PEI, noting that is not always beneficial (Davis and Nichols, 2016). This study confirms our hypothesis since as seen in Figure 1, emotional attention is negatively related to mood repair and resilience. This suggests that paying great attention to emotions can have harmful consequences, reducing an individual's ability to regulate emotions and limits the perception of benefits from adverse events (Guil et al., 2019).

Therefore, as observed, paying close attention to emotional states would impair the ability to repair negative emotions and recovering from dire events. However, if emotional attention is accompanied by an adequate ability to discriminate emotions, mood repair will not be affected, and in turn, it will no limit personal growth after experiencing these situations. This suggests that not all dimensions of PEI act in the same way as protective factors.

Although there is little literature on this topic, these findings are consistent with studies that show that the profile of people with better levels of psychological adaptation is characterized by low-moderate scores in emotional attention and high scores in emotional clarity and mood repair, suggesting that extreme levels of emotional attention are generally associated with emotional maladjustment (Extremera and Fernández-Berrocal, 2006). In this sense, if only this component of emotional intelligence is enhanced, it will affect the recovering from adverse situations. Therefore, the effectiveness of psychological interventions or therapeutic practices with a high attentional component, such as emotional consciousness or mindfulness, must be reviewed (Cebolla et al., 2017).

In other words, emotional regulation and strengthening after adversity will be affected if emotional attention is not accompanied by an adequate ability to discriminate emotional states (Cejudo et al., 2016; Merchán-Clavellino et al., 2019, 2020). Thus, if interventions aim to promote adjustment and psychological well-being, the dimensions of PEI should work complementary to avoid unwanted effects.

We suggest that clinical practice with these types of patients assess levels of emotional attention. If maladjustment is detected, a complete psycho-emotional intervention should be enhanced. However, in women with adequate levels of attention, we would only need to intervene in emotional regulation. Ultimately, this would imply a more personalized intervention design to the needs and more cost-effective in therapeutic practices.

To the best of our knowledge, this research increases the learning to date since it provides the processes through which PEI can act as a co-vitality factor, or on the contrary, lead to side effects in breast cancer survivors. Thus, it offers a more holistic view of socio-emotional variables that influence resilience indirectly, to promote the quality of life of breast cancer survivors.

This research is not without limitations, such as the use of self-report in data collection, since this type of information collection can generate biased responses (due to social desirability, halo effect, etc.), so that the results could be explained partly due to the variance bias of the common method. Besides, the causal interpretation is problematic because it is a cross-sectional study.

Future research should establish moderation models to know which levels of this variable are adaptive or not. Even a more detailed analysis of TMMS-24 in its attentional dimension would be interesting, for example, analyzing which items are more associated with adverse or maladaptive results and which elements would be more associated with adaptive results. Furthermore, it would be of interest to test this model in other types of cancer, even in patients with treatment, it will facilitate the generalization of our results throughout the oncological population. The medical characteristics of cancer should also be taken into account in other studies because it will allow to reveal possible variations in the mediation model and will understand how they influence the reaction to the disease, promoting or inhibiting a resistant response. Also, incorporating other variables, such as the social and interpersonal dimensions of emotional intelligence, into the compression of this mediation, would be interesting.

## Data Availability Statement

The raw data supporting the conclusions of this article will be made available by the authors, without undue reservation.

## Ethics Statement

The studies involving human participants were reviewed and approved by e-Health for Quality of Life and Health promotion in Breast Oncology PIN0109-2018. ETHICS COMMITTEE MINISTRY OF HEALTH (JJVM/apg). The patients/participants provided their written informed consent to participate in this study.

## Author Contributions

RG developed the project design and data analysis, data preparation, coding and writing the manuscript, and approved the final version of the manuscript for submission. PR-G contributed to project design, survey creation, performed the collection, the data analysis, interpretation, and writing of manuscript. AM-C contributed to the interpretation, writing of the manuscript, and approved the final version of the manuscript for submission. LM-S, AZ, and RG-M contributed to project design and data analysis, data preparation, coding and interpretation, and writing the manuscript. All authors contributed to the article and approved the submitted version.

## Conflict of Interest

The authors declare that the research was conducted in the absence of any commercial or financial relationships that could be construed as a potential conflict of interest.

## References

[B1] Ahoiee K. Faramarzi M. Hassanzadeh R. (2017). Psychological well-being of patients with breast cancer and its relationship with emotional intelligence. J. Babol Univ. Med. Sci. 19, 20–27. 10.22088/jbums.19.8.20

[B2] Amirifard N. Payandeh M. Aeinfar M. Sadeghi M. Sadeghi E. Ghafarpor S. (2017). A survey on the relationship between emotional intelligence and level of depression and anxiety among women with breast cancer. Int. J. Hematol. Oncol. Stem Cell Res. 11, 54. 28286616 PMC5338283

[B3] Baudry A. S. Lelorain S. Mahieuxe M. Christophe V. (2018). Impact of emotional competence on supportive care needs, anxiety and depression symptoms of cancer patients: a multiple mediation model. Support. Care Cancer 26, 223–230. 10.1007/s00520-017-3838-x 28779370

[B4] Brandão T. Schulz M. S. Matos P. M. (2017). Psychological adjustment after breast cancer: a systematic review of longitudinal studies. Psychooncology 26, 917–926. 10.1002/pon.4230 27440317

[B5] Brandão T. Tavares R. Schulz M. S. Matos P. M. (2016). Measuring emotion regulation and emotional expression in breast cancer patients: a systematic review. Clin. Psychol. Rev. 43, 114–127. 10.1016/j.cpr.2015.10.002 26520599

[B6] Burga I. Sánchez T. (2016). Inteligencia Emocional y Resiliencia en Pacientes Con Cáncer de Mama en el HNGAI-EsSalud de Lima, 2016. Doctoral Dissertation, Thesis de Licenciatura. Universidad Peruana Unión, Lima.

[B7] Catherine C. Camellia V. Husada M. S. Loebis B. Effendy E. Amin M. M. (2019). Affective psychopathology towards the quality of life of breast cancer patients with radiotherapy in Medan, Indonesia. Open Access Macedonian J. Med. Sci. 7, 1456. 10.3889/oamjms.2019.313 31198454 PMC6542408

[B8] Cebolla A. Demarzo M. Martins P. Soler J. Garcia-Campayo J. (2017). Unwanted effects: is there a negative side of meditation? A multicentre survey. PLoS One 12:e183137. 10.1371/journal.pone.0183137 28873417 PMC5584749

[B9] Cejudo J. López-Delgado M. L. Rubio M. J. (2016). Inteligencia emocional y resiliencia: su influencia en la satisfacción con la vida en estudiantes universitarios. Anuario Psicol. 46, 51–57. 10.1016/j.anpsic.2016.07.001

[B10] Davis S. K. Nichols R. (2016). Does emotional intelligence have a “dark” side? A review of the literature. Front. Psychol. 7, 1316. 10.3389/fpsyg.2016.01316 27625627 PMC5003940

[B11] Deshields T. L. Heiland M. F. Kracen A. C. Dua P. (2016). Resilience in adults with cancer: development of a conceptual model. Psychooncology 25, 11–18. 10.1002/pon.3800 25787828

[B12] European Cancer Information System (ECIS) (2018). Estimates of Cancer Incidence and Mortality in 2018, for all Countries. Retrieved from: https://ecis.jrc.ec.europa.eu (accessed July 4, 2020).

[B13] Extremera N. Fernández-Berrocal P. (2002). Relation of perceived emotional intelligence and health-related quality of life of middle-aged women. Psychol. Rep. 91, 47–59. 10.2466/pr0.2002.91.1.47 12353803

[B14] Extremera N. Fernández-Berrocal P. (2006). Emotional intelligence as predictor of mental, social, and physical health in university students. Span. J. Psychol. 9, 45–51. 10.1017/S1138741600005965 16673622

[B15] Fernández-Berrocal P. Alcaide R. Domínguez E. Fernández-McNally C. Ramos N. S. Ravira M. (1998). Adaptación al castellano de la escala rasgo de metaconocimiento sobre estados emocionales de Salovey et al.: datos preliminares. Libro Actas Cong Eval Psicol. 1, 83–84.

[B16] Fernández-Berrocal P. Cabello R. Castillo R. Extremera N. (2012). Gender differences in emotional intelligence: the mediating effect of age. Behav Psychol. 20, 77–89.

[B17] Furlong M. Dowdy E. Carnazzo K. Bovery B. L. Kim E. (2014). Covitality: Fostering the Building Blocks of Complete Mental Health. Communique 42, 1–28.

[B18] Gallagher M. W. Long L. J. Richardson A. D'Souza J. M. (2019). Resilience and Coping in Cancer Survivors: The Unique Effects of Optimism and Mastery. Cognit. Ther. Res. 43, 32–44. 10.1007/s10608-018-9975-9 31223177 PMC6586435

[B19] García Monzón L. Navarro Machado V. (2017). Factores moduladores de resiliencia en pacientes diagnosticadas con cáncer de mama. Revista Finlay 7, 250–259.

[B20] García-Maroto Fernández S. López Delgado M. L. Latorre Postigo J. M. (2015). Ansiedad, resiliencia e inteligencia emocional percibida en un grupo de mujeres con cáncer de mama. Ansiedad Estrés 21, 115–125.

[B21] Gómez-Baya D. Mendoza R. (2018). Trait emotional intelligence as a predictor of adaptive responses to positive and negative affect during adolescence. Front. Psychol. 9, 2525. 10.3389/fpsyg.2018.02525 30618950 PMC6297835

[B22] González A. E. M. Piqueras J. A. Linares V. R. (2010). Inteligencia emocional en la salud física y mental. Electr J. Res. Educ. Psychol. 8, 861–890.

[B23] Guil R. Gómez-Molinero R. Merchan-Clavellino A. Gil-Olarte P. Zayas García A. (2019). Facing anxiety, growing up. Trait emotional intelligence as a mediator of the relationship between self-esteem and pre-university anxiety. Front. Psychol. 10, 567. 10.3389/fpsyg.2019.00567 30930824 PMC6429289

[B24] Guil R. Zayas A. Gil-Olarte P. Guerrero C. González S. Mestre J. M. (2016). Bienestar psicológico, optimismo y resiliencia en mujeres con cáncer de mama. Psicooncología 13, 127 10.5209/rev_PSIC.2016.v13.n1.52492

[B25] Hayes A. F. (2013). Introduction to Mediation, Moderation, and Conditional Process Analysis: A Regression-Based Approach. New York, NY: The Guilford Press.

[B26] Hermosilla-Ávila A. Sanhueza-Alvarado O. (2015). Control emocional, felicidad subjetiva y satisfacción vital relacionados al afrontamiento y adaptación en personas con cáncer avanzado. Ciencia Enfermería 21, 11–21. 10.4067/S0717-95532015000100002

[B27] Lizeretti N. P. Extremera N. Rodríguez A. (2012). Perceived emotional intelligence and clinical symptoms in mental disorders. Psychiatric Q 83, 407–418. 10.1007/s11126-012-9211-9 22350130

[B28] Mahdavi A. Taghizadeh M. E. Isazadeh S. Rezaei A. Eghtedarnejad S. Sepehryeganeh S. (2015). Effectiveness of emotional intelligence components on social adjustment and social intimacy of women with breast cancer. Mediterr. J. Soc. Sci. 6, 59 10.5901/mjss.2015.v6n6s6p59

[B29] Markovitz S. E. Schrooten W. Arntz A. Peters M. L. (2015). Resilience as a predictor for emotional response to the diagnosis and surgery in breast cancer patients. Psycho Oncol. 24, 1639–1645 10.1002/pon.3834 25967598

[B30] Marroquín B. Czamanski-Cohen J. Weihs K. L. Stanton A. L. (2016). Implicit loneliness, emotion regulation, and depressive symptoms in breast cancer survivors. J. Behav. Med. 39, 832–844. 10.1007/s10865-016-9751-9 27287618 PMC5014704

[B31] Merchán-Clavellino A. Alameda-Bailén J. R. García A. Z. Guil R. (2019). Mediating effect of trait emotional intelligence between the Behavioral Activation System (BAS)/Behavioral Inhibition System (BIS) and Positive and Negative Affect. Front. Psychol. 10:424. 10.3389/fpsyg.2019.00424 30890980 PMC6411706

[B32] Merchán-Clavellino A. Salguero-Alcañiz M. P. Guil R. Alameda-Bailén J. R. (2020). Impulsivity, emotional intelligence, and alcohol consumption in young people: a mediation analysis. Foods 9, 71. 10.3390/foods9010071 31936411 PMC7022743

[B33] Mestre J. M. Fernández-Berrocal P. (Coords.) (2007). Manual de Inteligencia Emocional. Madrid: Pirámide.

[B34] Miao C. Humphrey R. H. Qian S. (2017). A meta-analysis of emotional intelligence and work attitudes. J. Occup. Organ. Psychol. 90, 177–202. 10.1111/joop.12167

[B35] Molano-Tobar N. J. Varela P. E. V. (2017). Percepción acerca del cáncer de mama en un grupo de mujeres de un hospital en Popayán, Colombia. MHSalud 13, 1–14. 10.15359/mhs.13-2.5

[B36] Novella J. (2002). Adaptación de la Escala de Resiliencia de Wagnild y Young en la ciudad de Lima. Revista Científica Universidad Mayor de San Marcos. Lima-Perú.

[B37] Padilla-Ruiz M. Ruiz-Román C. Pérez-Ruiz E. Rueda A. Redondo M. Rivas-Ruiz F. (2019). Clinical and sociodemographic factors that may influence the resilience of women surviving breast cancer: cross-sectional study. Support. Care Cancer 27, 1279–1286. 10.1007/s00520-018-4612-4 30607680

[B38] Pérez-González J. C. Yáñez S. Ortega-Navas C. Piqueras J. A. (2020). Educación emocional en la educación para la salud: cuestión de salud pública. Clín. Salud. 31, 127–136. 10.5093/clysa2020a7

[B39] Petrides K. V. (2010). Trait emotional intelligence theory. Ind. Organ. Psychol. 3, 136–139. 10.1111/j.1754-9434.2010.01213.x

[B40] Petrides K. V. Mikolajczak M. Mavroveli S. Sanchez-Ruiz M. J. Furnham A. Pérez-González J. C. (2016). Developments in trait emotional intelligence research. Emotion Rev. 8, 335–341. 10.1177/1754073916650493

[B41] Preacher K. J. Hayes A. F. (2008). Asymptotic and resampling strategies for assessing and comparing indirect effects in multiple mediator models. Behav. Res. Methods 40, 879–891. 10.3758/BRM.40.3.879 18697684

[B42] Resurrección D. M. Salguero J. M. Ruiz-Aranda D. (2014). Emotional intelligence and psychological maladjustment in adolescence: a systematic review. J. Adolesc. 37, 461–472. 10.1016/j.adolescence.2014.03.012 24793394

[B43] Rey L. Extremera N. Trillo L. (2013). Exploring the relationship between emotional intelligence and health-related quality of life in patients with cancer. J. Psychosoc. Oncol. 31, 51–64. 10.1080/07347332.2012.703770 23311971

[B44] Rider Mundey K. Nicholas D. Kruczek T. Tschopp M. Bolin J. (2019). Posttraumatic growth following cancer: the influence of emotional intelligence, management of intrusive rumination, and goal disengagement as mediated by deliberate rumination. J. Psychosoc. Oncol. 37, 456–477. 10.1080/07347332.2018.1514449 30595107

[B45] Ruiz-González P. Zayas A. Morales-Sánchez L. Gil-Olarte P. Guil R. (2019). Resiliencia como predictora de depresión en mujeres con cáncer de mama. Int. J. Dev. Educ. Psychol. 1, 75–84. 10.17060/ijodaep.2019.n1.v4.1511

[B46] Salovey P. Mayer J. D. (1990). Emotional intelligence. Imagin. Cogn. Pers. 9, 185–211. 10.2190/DUGG-P24E-52WK-6CDG

[B47] Salovey P. Mayer J. D. Goldman S. L. Turvey C. Palfai T. P. (1995). “Emotional attention, clarity, and repair: exploring emotional intelligence using the Trait Meta-Mood Scale,” in Emotion, Disclosure, and Health, ed J. W. Pennebaker (Washington, DC: American Psychological Association), 125–154.

[B48] Schmidt J. E. Andrykowski M. A. (2004). The role of social and dispositional variables associated with emotional processing in adjustment to breast cancer: an internet-based study. Health Psychol. 23, 259. 10.1037/0278-6133.23.3.259 15099166

[B49] Stanton A. L. Bower J. E. (2015). “Psychological adjustment in breast cancer survivors,” in Improving Outcomes for Breast Cancer Survivors: Perspectives on Research Challenges and Opportunities, ed P. A. Ganz (Cham: Springer International Publishing), 231–242.

[B50] Wagnild G. Young H. (1993). Development and psychometric evaluation of the Resilience Scale. J. Nurs. Meas. 1, 165–178. 7850498

[B51] World Health Organization (2014). World Cancer Report 2014. Retrieved from: https://www.drugsandalcohol.ie/28525/1/World%20Cancer%20Report.pdf. 10.30875/9f925144-en

[B52] Zayas A. Guil R. Guerrero C. Gil-Olarte P. Mestre J. M. (2016). “Resilience, optimism, and depression in caregivers of diabetic children,” in QUAESTI-Virtual Multidisciplinary Conference (No. 1) (Zilina).

[B53] Zayas A. Morales L. Ruiz-González P. Guil R. (2019). Estrategias de afrontamiento y su capacidad predictiva en los niveles de resiliencia en una muestra de mujeres con cáncer de mama. Int. J. Dev. Educ. Psychol. 5, 279–290. 10.17060/ijodaep.2019.n1.v5.1598

